# Retention of Urine in the Child

**Published:** 1901-06-15

**Authors:** 


### RETENTION OF URINE IN THE CHILD.

In a recently-delivered clinical lecture on retention of urine Mr. Christopher Heath[1] referred to the causes which might lead to this condition in children. One of these is a tight foreskin. When this occurs the infant very soon after birth is noticed not to be passing urine or only to be passing very little, and possibly the foreskin may be noticed to be what is termed "ballooned." The treatment is simply to dilate the orifice with a pair of forceps, or one may circumcise, but in Mr. Heath's opinion dilating the orifice does just as well. Later in life retention in a child may be caused by a calculus in the urethra. The narrowest point in this passage is at the meatus, and it is near the meatus that a stone is likely to lodge if it lodges at all. For the removal of the stone it may be necessary to make a nick in the orifice, and if this should have to be done the nick should be made downwards where it can do no harm; if the cut be made upwards it will go into the vascular tissue of the glans. There is one cause of retention against which one has to be on one's guard, and that is the possibility that a thread may have been tied round the penis. This happens generally in children who have been punished for wetting the bed, the child in desperation tying a thread round his penis to prevent this occurrrence. This form of strangulation of the penis may, however, happen in other ways; and Mr. Heath relates a case of a child who was brought to his house with the glans penis hanging merely by a few threads of cellular tissue, on snipping through which with a pair of scissors he found that the strangulation had been caused by a long hair which was wound round the part.

[1] Lancet, June 8.

